# Unveiling Thermal Degradation and Fire Behavior of 110 kV Ultra-High-Voltage Flame-Retardant Cable Sheath After Thermal Aging

**DOI:** 10.3390/polym17091273

**Published:** 2025-05-06

**Authors:** Yaqiang Jiang, Wei He, Xinke Huo, Xuelian Lu, Kaiyuan Li, Fei Xiao

**Affiliations:** 1 Key Laboratory of Fire Protection and Retardant Technology, Ministry of Emergency Management, Chengdu 610036, China; jiang.yaqiang@scfri.cn; 2 Sichuan Fire Research Institute of MEM, Chengdu 610036, China; 3 School of Safety Science and Emergency Management, Wuhan University of Technology, Wuhan 430070, China; wei_he@whut.edu.cn (W.H.); xinke.huo@whut.edu.cn (X.H.); 4 Shenzhen Research Institute, Wuhan University of Technology, Shenzhen 518057, China; xuelianlu@126.com

**Keywords:** flame-retardant cable sheath, thermal aging, thermal degradation, fire behavior

## Abstract

To evaluate the fire safety of 110 kV ultra-high-voltage flame-retardant polyvinyl chloride (PVC) cables in the service process, the effects of thermal aging on the pyrolysis and combustion behavior of the cable sheaths were studied using thermogravimetric (TG), limiting oxygen index (LOI), UL-94 vertical burning, cone calorimeter, open flame, and muffle furnace tests. The results showed that thermal aging causes a slight decrease in the LOI value of the cable sheath (28.3% vs. 28.5%), but it also passed the UL-94 V-0 test. The butane torch test showed that the cable sheath was more easily ignited after aging; however, a better char layer was formed in the later stage of burning, which led to a longer failure time. Interestingly, the aging treatment prolonged the ignition time of the cable sheaths and reduced the peak heat release rate (pHRR) and total heat release (THR) by 17.5% and 24.4%, respectively, in the cone calorimeter test, indicating that aging resulted in a reduction in the fire hazard of the cable sheaths. Moreover, aging mechanisms were proposed based on the composition and structural evolution of the cable sheaths. In summary, this work comprehensively evaluated the fire hazard of 110 kV ultra-high-voltage cables and provided theoretical support for the formulation improvement, durability enhancement, and fire protection design of cable sheath materials.

## 1. Introduction

The safe operation of ultra-high-voltage (UHV) cables in utility tunnels is crucial to meeting the growing demand for electricity [1]. However, UHV cables pose a great fire safety hazard because they contain a large amount of combustible polymer materials (i.e., outer sheath and insulation) and are affected by complex electrical, thermal, and environmental factors [2,3]. Commercial UHV cables usually introduce flame retardants into the outer sheath to protect the core from fire damage; however, their thermal stability and combustion performance may deteriorate due to long-term service in high-temperature utility tunnels (mainly Joule heat) [4].

Numerous studies have examined the pyrolytic, combustion, and fire spread behaviors of cables under different cable types [5], arrangements [6], environmental pressures [7], and external heat or fire sources [8]. However, these works mainly focused on the overall performance of unaged cables, ignoring the fact that thermal aging may cause changes in the chemical composition and structure of cables, resulting in different physicochemical properties [9], including thermal degradation behavior [10,11,12], electrical properties [13], and mechanical properties [14]. It was found that thermal aging did not completely deplete the antioxidants in the cross-linked polyethylene (XLPE) cable, but it led to re-crystallization and re-degassing of the cables, affecting the behavior of space charge and charge carrier trapping [13]. Moreover, the effect of aging on the mechanical performance of various PVC cable sheaths was also investigated, and the results confirmed that aging led to the loss of certain mechanical properties [15]. Similarly, as the PVC cables aged, their relative hardness and plasticizer content decreased [16].

Thermal aging induces structural changes in the polymer matrix in the cable sheaths, such as chain scission and cross-linking [17,18], leading to a certain degree of deterioration of the cable, which may affect its pyrolysis and fire behavior [19]. Unfortunately, this effect has not yet attracted much attention, and even contradictory results have emerged in some studies [20]. Some studies have demonstrated that the pHRR of PVC cables increased while their ignition time decreased following thermal aging, indicating a deterioration in fire retardancy [21]. On the other hand, it has also been pointed out that thermal aging increased the activation energy of polyethylene cables, making them more difficult to pyrolyze and more resistant to catching fire compared to unaged cables [22]. The influence of thermal aging temperatures on the pyrolysis and combustion of PVC cables was investigated, and it was found that the LOI values increased with the increase in aging time or aging temperature [4]. Similarly, the early fire risks of the polychloroprene rubber-based cable sheath decreased with the increase in aging years [23], but the fire risks during the mid and late periods, as well as fire intensity and severity, increased with the increase in aging years. This may be attributed to the fact that the heat aging process removes some of the more volatile components in the cable sheaths, resulting in a decrease in the emission of combustible gases during burning and therefore a decrease in flammability [24].

Obviously, there is still some controversy about the impact of thermal aging on cable fire risk, which may be related to factors such as cable sheath composition, aging time, and aging temperature. Furthermore, several parameters could be used to evaluate the fire risks associated with cable sheaths. These include ignition time, which assesses ignition risk; flame spread behavior, which evaluates fire growth risk; heat release rate, which measures heat release risk; and dripping behavior, which may lead to secondary fires [19]. Among these factors, the relationship between thermal aging and ignition and heat release has garnered more attention, whereas flame spread and fire resistance behavior have received comparatively less focus. To comprehensively assess the impact of thermal aging on the fire risk associated with cables, it is essential to further investigate the thermal stability, flame spread, and fire resistance behaviors of cable sheaths both before and after aging.

In this study, the thermal accelerated aging method was applied to treat 110 kV flame-retardant PVC cable sheaths. The effects of thermal aging on the thermal stability, pyrolysis, fire retardancy, and fire resistance of the PVC cable sheath were evaluated using thermogravimetric analysis (TGA), LOI, UL-94 vertical burning test, cone calorimeter, and fire resistance tests. Meanwhile, the structural changes in the cable sheaths were analyzed using Fourier transform infrared (FTIR) spectroscopy to propose a possible aging mechanism.

## 2. Experimental Procedure

### 2.1. Materials

The 110 KV ultra-high-voltage cable (ZC-YJLW0264/110kV) used in this study was supplied by Sunway Co. Ltd., Leshan, China, and the flame-retardant cable sheath is composed of PVC resin, flame retardant, plasticizer, stabilizer, etc. Before testing, all samples were treated in an oven at 60 °C for 24 h to remove moisture.

### 2.2. Thermal Aging

The cable sheath thermal aging test was used to evaluate the deterioration of the physical properties of the sheath after long-term use at high temperatures. According to the thermal aging method specified in GB/T2951.12-2008, the cable sheath was aged by placing it in an air circulation oven at an aging temperature of 135 °C for 14 days. The unaged and aged cable sheaths are denoted as S1 and S2, respectively.

### 2.3. Characterization

#### 2.3.1. LOI Test

The limiting oxygen index (LOI) values of the cable sheaths were determined using a fully automatic oxygen index tester (YZS-8A, Xinsheng Zhuorui Technology Co., Ltd., Beijing, China) according to the ASTM D2863-19 standard. The sample size was 100 mm × 6.5 mm × 5.2 mm.

#### 2.3.2. UL-94 Test

A vertical combustion test of the cable sheaths with a size of 100 mm × 6.5 mm × 5.2 mm was carried out using a 2 cm blue butane flame to evaluate its flammability, fire spread, and self-extinguishing properties.

#### 2.3.3. Contact Angle Test

A contact angle tester (Dingsheng JY-82C) was used to measure the hydrophobicity of the cable sheath before and after thermal aging treatment. Before the test, the impurities on the surface of the sheath were cleaned with deionized water and dried to ensure the accuracy of the test results.

#### 2.3.4. Cone Calorimeter Test

The heat release and smoke characteristics of the cable sheaths before and after aging treatment were tested using a cone calorimeter (Motis Fire Technology Co., Ltd., Kunshan, China) in accordance with the ISO 5660-1:2016 standard. The radiant heat flux was 50 kW/m^2^ and the distance between the heater and the sample was 25 mm.

#### 2.3.5. Muffle Furnace Test

The high temperature stability of the cable sheaths before and after aging treatment was tested using a muffle furnace (Shaoxing Shangyu Daoxu Kexi Instrument Factory, Shaoxing, China). The samples before and after aging were heat-treated at different temperatures (200, 250, 300, 350, 400, and 500 °C) for 10 min, and the morphology, composition, and mass changes of the samples were recorded.

#### 2.3.6. TGA Test

The PVC sheath is the main combustible material of the cable, so its pyrolysis characteristics play a key role in analyzing the fire hazard of the cable. Thermogravimetric analysis (TGA) was performed using a simultaneous thermer analyzer STA 8000 (PerkinElmer Inc., Waltham, MA, USA). Then, 9–10 mg samples were heated from 30 to 800 °C at a heating rate of 10 °C/min under nitrogen conditions.

#### 2.3.7. Open Flame Test

The open flame test was carried out on a home-made bench using a butane torch [25]. The distance between the butane torch nozzle and the cable sheath was 7 cm. The flame temperature of the butane torch was experimentally measured to be in the range of 1100–1200 °C. Thermocouples were connected to the back of the cable sheaths to record the real-time temperature over time. At the same time, an infrared camera was used to observe the combustion behavior of the cable sheaths.

#### 2.3.8. FTIR Test

FTIR was carried out using a Nicolet IS50 FTIR spectrometer (Thermo Scientific, Waltham, MA, USA) with a scanning resolution of 4 cm^−1^ to obtain the FTIR spectra of the cable sheaths in the range of 400–4000 cm^−1^.

#### 2.3.9. SEM Test

A scanning electron microscope (TESCAN MIRA LMS, Brno, Czech Republic) was used to characterize the surface morphology of the cable sheaths before and after aging treatment and to observe the morphology of the residual char.

## 3. Results and Discussion

### 3.1. Morphological Analysis and Hydrophilicity Analysis

**Figure 1:**
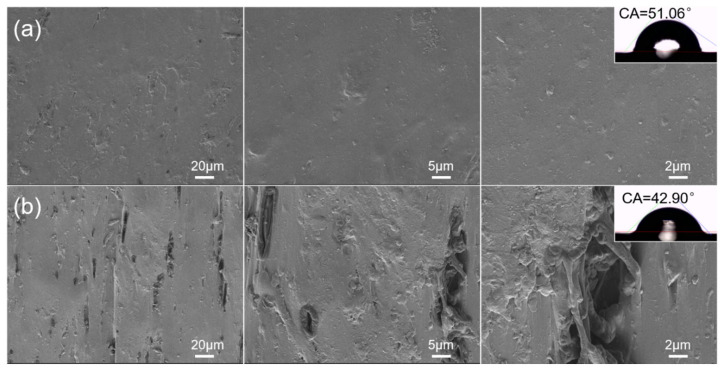
Surface morphology SEM images and CAs of (**a**) unaged (S1) and (**b**) aged (S2) cable sheaths.

The surface microstructure of the cable sheaths before and after thermal aging treatment is shown in Figure 1. The surface of the cable sheath before aging is relatively smooth, with only a few tiny defects or pits, no obvious cracks or large holes, a uniform texture and structure, and good material integrity, indicating that its structure is tight. However, after aging, the surface roughness of the cable sheath increases significantly, with granular protrusions, holes, and cracks. The difference in surface morphology before and after aging treatment indicates that thermal aging may cause a certain degree of deterioration in the PVC cable sheath, which may be attributed to the rapid loss of the plasticizer, the migration of inorganic components, and the rearrangement and aggregation of PVC molecular chains by the annealing effect [26,27]. At the same time, obvious hardening after aging treatment is observed, which is attributed to the decrease in the flexibility of the cable sheath caused by the volatilization or migration of the plasticizer, thereby promoting the formation of surface holes and cracks [20]. These changes suggest that thermal aging affects the composition and distribution of materials inside the sheath, which may affect the pyrolysis and combustion behavior of the cable sheath [4].

The contact angles (CAs) of the cable sheaths before and after aging treatment are shown in Figure 1. The CA value of S1 is 51.06°. After thermal aging treatment, the CA value of the cable sheath (S2) decreases to 42.90°, a reduction of 16%. This phenomenon suggests that the hydrophilicity (or wettability) of the material surface has increased. This change may be due to the degradation of the molecular chain of the cable sheath during the thermal aging process, resulting in the breakage of the molecular chain and accompanied by an oxidation reaction, which forms more polar groups (e.g., carbonyl, hydroxyl) on the surface of the material [4,28], thereby increasing the affinity of the material for water molecules. In addition, thermal aging causes a significant increase in the roughness of the material surface. The cracks, holes, and uneven structure on the surface provide more effective contact areas for the adsorption of water molecules, thereby further reducing the CA value.

### 3.2. LOI and UL-94 Tests

**Figure 2:**
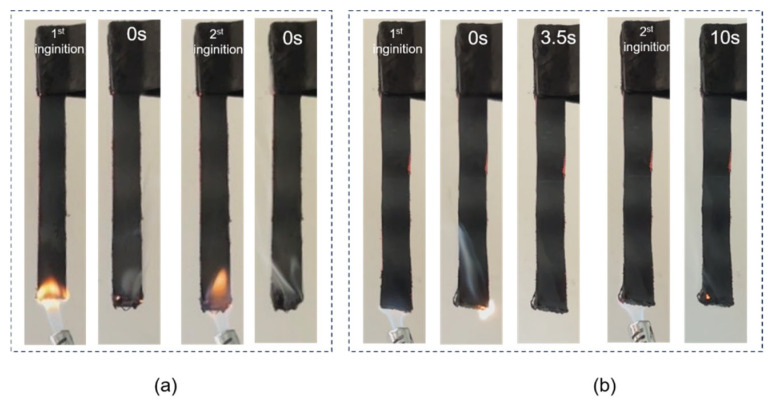
Fire behavior of (**a**) S1 and (**b**) S2 during the UL-94 test.

The LOI and UL-94 results of the cable sheaths before and after thermal aging are shown in Table 1. The LOI value of S1 is 28.5%, while after aging treatment, the LOI value of S2 is slightly reduced, to 28.3%. Meanwhile, all the cable sheaths reach the UL94 V-0 rating; S1 extinguishes quickly after leaving the fire, while the aged S2 continues to burn for about 3.5 s after the flame is removed (Figure 2). These results show that thermal aging leads to increased flammability of cable sheaths, which may be related to the migration of plasticizers and the oxidative degradation of the aged PVC cable sheath, resulting in an increase in cracks and holes in the material, increasing the diffusion channel of oxygen and accelerating the combustion of the material [4,23]. In addition, the volatilization or decomposition of flame retardants and the de-chlorination of PVC during aging may also lead to a decrease in the flame retardancy (in terms of LOI value) of the cable sheaths, as reported in previous literature [29].

### 3.3. Forced Flaming Combustion Test

**Figure 3:**
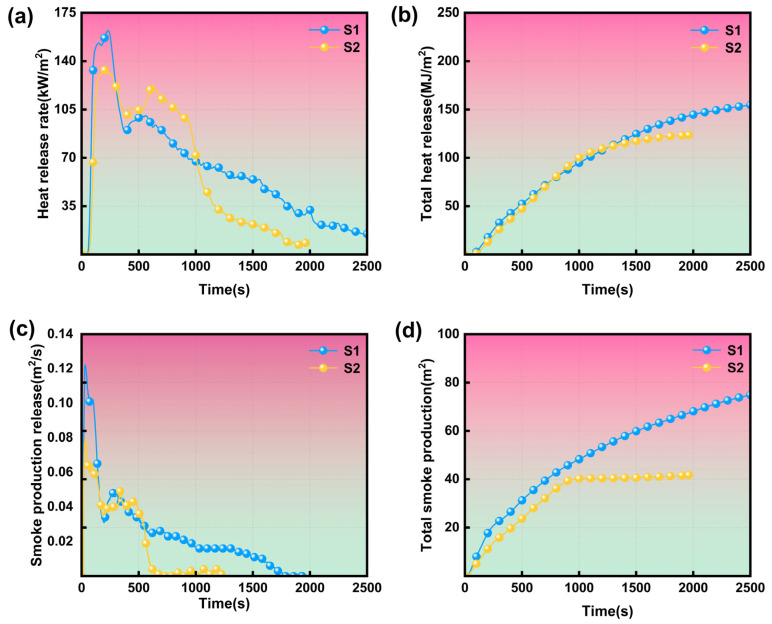
(**a**) HRR, (**b**) THR, (**c**) SPR, and (**d**) TSP curves of aged and unaged cable sheaths.

A cone calorimeter was applied to evaluate the effect of thermal aging on forced combustion behavior and fire retardancy of cable sheaths, and the relevant data are shown in Table 2 and Figure 3, including time to ignition (TTI), peak heat release rate (pHRR), total heat release (THR), peak smoke production rate (PSPR), and total smoke production (TSP).

The TTI is defined as the duration required for the surface of a material subjected to heating to attain the threshold of continuous combustion under a specified incident heat flux intensity [30]. The TTI serves as a valuable metric for evaluating and comparing the flammability and fire resistance of different materials; a reduced TTI signifies an increased fire hazard associated with the material. As indicated in Table 2, the TTI for S2 is marginally greater than that of S1, implying that thermal aging endows S2 with higher resistance to ignition compared to S1 when exposed to the high external heat flux, which is consistent with previous studies [19].

As illustrated in Figure 3a, the HRR of S1 and S2 exhibits a pronounced increase following ignition, reaching the first peak before gradually declining and subsequently rising again to a second peak. The first peak in HRR is attributed to the combustion of volatile gases generated from the PVC materials during the early stages of pyrolysis. As the pyrolysis process advances, the flame retardant absorbs heat and interacts with the polymer matrix, leading to the formation of a char layer. This layer impedes the pyrolysis and combustion of internal combustible gases, resulting in a gradual decline in HRR. However, as the experiment progresses, the char layer undergoes oxidation, expansion, or cracking, which facilitates the rapid release of pyrolysis gases through the fissures in the char layer, thereby producing a second peak in HRR [31]. In comparison to S1, the pHRR1 of S2 exhibited a reduction from 162.1 kW/m^2^ to 133.7 kW/m^2^, representing a decrease of approximately 18%. This decline may be attributed to the volatilization of small molecules, plasticizers, and other additives present in the cable during the aging process, which subsequently diminished the quantity of combustible components [32]. Conversely, pHRR2 increased from 100.5 kW/m^2^ to 122.6 kW/m^2^, reflecting an increase of around 22%. This rise may be linked to the migration and precipitation of flame retardants from the flame-retardant cable at an aging temperature of 135 °C, leading to a reduction in the flame retardant content and an increase in heat release [4].

As illustrated in Figure 3b, the THR for S2 is recorded at 123.7 MJ/m^2^, whereas S1 demonstrates a THR of 163.6 MJ/m^2^. The significantly higher THR of S1 indicates a more intense combustion process, characterized by greater combustion efficiency and increased heat output. This observation can be further elucidated by examining the combustion duration; S2 exhibits a combustion time of approximately 2000 s, in contrast to S1, which has a combustion time of about 2500 s. Given that THR is fundamentally the integral of the heat release rate (HRR) over time, the disparity in combustion duration accounts for the lower THR observed in S2 compared to S1.

As illustrated in Figure 3c, the SPR for S1 and S2 exhibits a rapid increase following ignition, culminating in the first peak. Subsequently, the SPR continues to ascend after a minor decline, ultimately reaching a second peak, a pattern that closely mirrors the behavior of the HRR. The maximum SPR recorded for sample S1 is 0.122 m^2^/s, with a TSP of 76.7 m^2^. In contrast, S2 demonstrates a maximum SPR of 0.079 m^2^/s and a TSP of 41.7 m^2^. The reduced smoke emission associated with S2 may be attributed to its higher char residue yield, suggesting a propensity for the formation of char residues during burning rather than the generation of volatiles.

Two critical engineering parameters, namely the flame growth rate index (FIGRA) and the maximum average rate of heat emission (MARHE), are employed to further assess the fire hazard of S1 and S2 [33,34]. FIGRA represents the maximum value of the ratio of HRR to the time required to reach that HRR as a standardized indicator for evaluating the fire risk and reaction to the fire class of electrical cables [35,36]. A higher FIGRA indicates faster flame spread and a higher fire risk of the material [37]. The FIGRA for S2 decreases from 1.33 for S1 to 0.92, suggesting that thermal aging contributes to the reduction of heat-related fire hazard. In addition, MARHE denotes the maximum value of the ratio of THR to the corresponding time, which can be used to comprehensively assess the thermal impact of the fire on the surroundings [38]. As shown in Table 2, the difference in MARHE values between S2 and S1 is very small (0.11 vs. 0.10), proving that the thermal aging of cables does not have a significant influence on MARHE.

**Figure 4:**
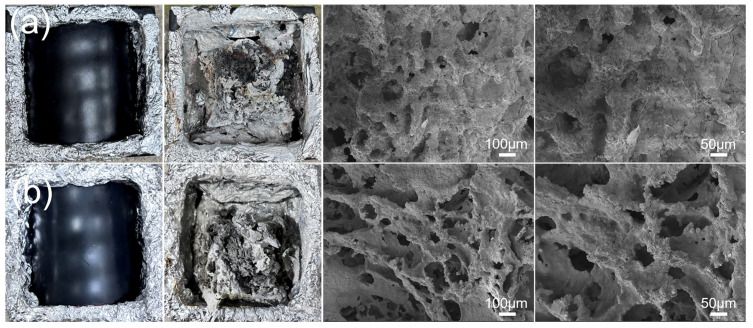
Digital photographs and SEM images of char residues of (**a**) S1 and (**b**) S2.

As shown in Figure 4, the flame-retardant PVC cable sheath forms protective char residues after the cone calorimeter test. The char layer effectively blocks heat and mass transfer, as well as flame propagation, to a certain extent. It delays combustion of the underlying matrix, thereby inhibiting the growth of HRR and THR. In comparison, S2 forms more carbonaceous layers, which may be attributed to the changes in the structure and composition of the cable sheath caused by aging. Meanwhile, SEM images (Figure 4) show that after aging, a more complex spatial structure is formed inside the carbonaceous layer, which may act as a thermal barrier and effectively inhibit the release of flammable volatiles.

### 3.4. Fire Resistance

**Figure 5:**
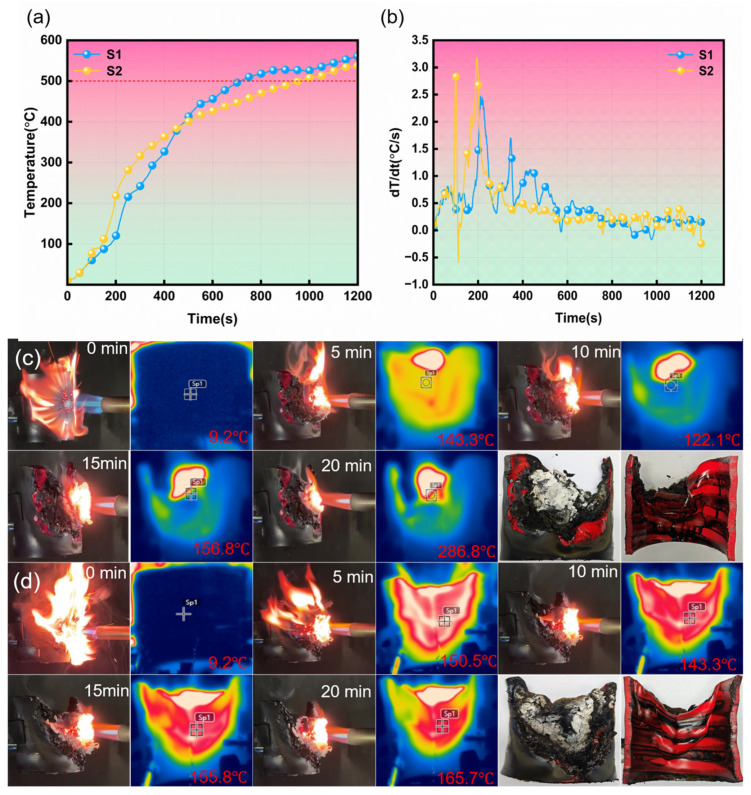
(**a**,**b**) Backside temperature of aged and unaged cable sheaths in the open flame test; digital photos and infrared images of (**c**) unaged and (**d**) aged cable sheaths in the open flame test.

When cables are exposed to severe external fire, their sheaths tend to catch fire and cause circuit systems to break or short-circuit [39]. To evaluate the effect of thermal aging on the fire resistance of 110 kV flame-retardant PVC cables, butane torch tests were performed on the sheaths of the cables before and after aging. Figure 5a shows the change in the backside temperature of the cable sheathing over time, as measured by the thermocouples. At ignition, there is no significant difference in the temperatures of S1 and S2. After approximately 150 s, the temperature of S2 rises rapidly and surpasses that of S1. This may be attributed to the reduction in the initial thermal stability of the cable sheath due to thermal aging. Around 460 s, the temperatures of S1 and S2 equalize, after which the temperature of S1 increases sharply. It is clear that the time required for S2 to reach 500 °C is delayed by more than 100 s compared to S1, suggesting a higher fire safety. Figure 5b depicts the rate of change of the cable sheath temperature over time. Obviously, within 60 to 200 s, the heating rate of S2 is significantly greater than that of S1, while within 300 to 770 s, the opposite pattern is observed. This result indicates that although S2 is more susceptible to ignition, it exhibits better fire resistance during sustained combustion compared to S1.

Figure 5c,d illustrate the combustion behavior of the cable sheaths when subjected to a butane torch. Visual observation shows that S2 burns violently during ignition. After burning for 5 min, the high-temperature area in the infrared image nearly encompasses the entire material, with an infrared temperature recorded at 150.5 °C. After burning for 10 min, a char layer forms on the surface of the cable sheath, and the infrared temperature decreases to 143.3 °C. Subsequently, the temperature increases further, and after 20 min, the infrared temperature reaches 165.7 °C, and the char layer appears relatively fragmented. For S1, at ignition, the flame is weaker, and the burning area is relatively concentrated. After burning for 5 min, the infrared temperature measures 143.3 °C, which is lower than that of S2. After 10 min, the infrared temperature decreases to 122.1 °C. However, after 20 min, the infrared temperature for S1 is significantly higher than that of S2, at 286.8 °C, and notches begin to appear. The trends observed in the infrared images are consistent with the temperature changes recorded by the thermocouples. Comparing the char layers on the surfaces of S1 and S2 after 20 min, it is revealed that the char layer of S1 is denser, while the char layer of S2 is more broken and exhibits a relatively fragile structure, which is consistent with the SEM images of the residual char.

These results indicate that S2 absorbs heat more readily during the initial stages of burning, leading to a more rapid increase in temperature. This phenomenon may be attributed to the thermal aging treatment, which caused the loss of plasticizers and heat stabilizers within the cable, thereby decreasing its heat resistance. As a result, heat diffuses more quickly, intensifying the burning process. In the later stages of burning, the temperature of S1 exceeds that of S2. This discrepancy may arise from the formation of a cross-linked structure in S2 during the high-temperature oxidative decomposition process, which impedes the transfer and diffusion of heat. In addition, S2 possesses an unstable porous carbon layer that is detrimental to pyrolysis and combustion [4]. Its high porosity and fragmented structure diminish the heat conduction pathway, whereas S1 features a denser carbon layer with superior thermal conductivity, resulting in a higher backside temperature of the cable sheaths.

### 3.5. Thermal Stability Analysis

**Figure 6:**
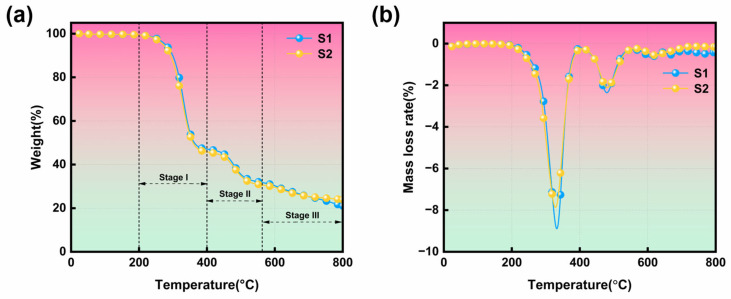
(**a**) TG and (**b**) DTG curves of unaged and aged cable sheaths.

The TG and DTG curves of the cable sheath before and after heat aging treatment are shown in Figure 6, and the relevant parameters are summarized in Table 3. As illustrated in Figure 6, the DTG curve of the cable sheath exhibits three primary peaks. This study posits that the process of PVC can be categorized into three stages. The first stage, occurring at 200–400 °C, is associated with the volatilization of the plasticizer and the dehydrochlorination reaction [4,12,29]. The second stage, which occurs within the temperature range of 400–570 °C, is attributed to the cleavage and pyrolysis of polyenes containing conjugated double bonds, as well as the cyclization and fragmentation of the chain, leading to the formation of linear or cyclic low hydrocarbons [40]. The third stage primarily involves the slow degradation of the remaining residue at 570–800 °C [4,12]. The thermogravimetric analysis of the cable sheath focuses mainly on the first two processes. The weight loss rate of S1 in the first stage is 45.7%, and the weight loss rate in the second stage is 22.4%, resulting in a final residual mass of 21.0%. In comparison, the weight loss rate of S2 in the first stage increased to 47.1%, and the weight loss rate in the second stage was nearly equal to that of S1 (22.2%). The final residual mass of S2 increased to 23.9% compared to S1, which may be attributed to the volatilization of combustible substances during the aging process [40]. From the data presented, it is evident that thermal aging primarily affects the first decomposition process, which is attributed to dehydrochlorination, volatility or decomposition of plasticizers, and the intensification of chain scission and cross-linking of polymers [4]. The relevant parameters listed in Table 3 indicate that the T_5%_ value of S2 decreased from 277.51 °C for S1 to 271.53 °C for S2. Additionally, the residual yield of S2 (23.9%) was greater than that of S1 (21.0%), and the DTG_peak1_ of S2 was lower than that of S1. This phenomenon suggests that although the thermal stability of S2 diminished and it began to pyrolyze at a lower temperature, which is consistent with the experimental results of the open flame, S1 experienced a more complete and intense pyrolysis process than S2.

Changes in the chemical composition, structure, and additives of polymers during thermal aging may account for the differing thermal degradation behaviors of cables before and after aging [12]. In the initial stages of pyrolysis, PVC cable sheaths contain a higher concentration of plasticizers and stabilizers. These small organic molecules may volatilize or decompose during the thermal aging process, resulting in the material becoming hard and brittle. This degradation decreases the thermal stability of the material, making it more susceptible to combustion and cracking. Concurrently, the carbon content of the sheath material increases, facilitating the formation of residual carbon after pyrolysis. Subsequently, the aged PVC cable sheath undergoes thermal oxidative decomposition and a cross-linking reaction, resulting in a more stable structure that reduces the degradation rate of the cable sheath, produces a greater quantity of solid carbonized products, and increases the residual char content [4]. Additionally, PVC cable sheaths contain a significant amount of inorganic fillers, which decompose at high temperatures to release water vapor and CO_2_, inhibiting pyrolysis and promoting carbonization [41].

### 3.6. Aging Mechanism

**Figure 7:**
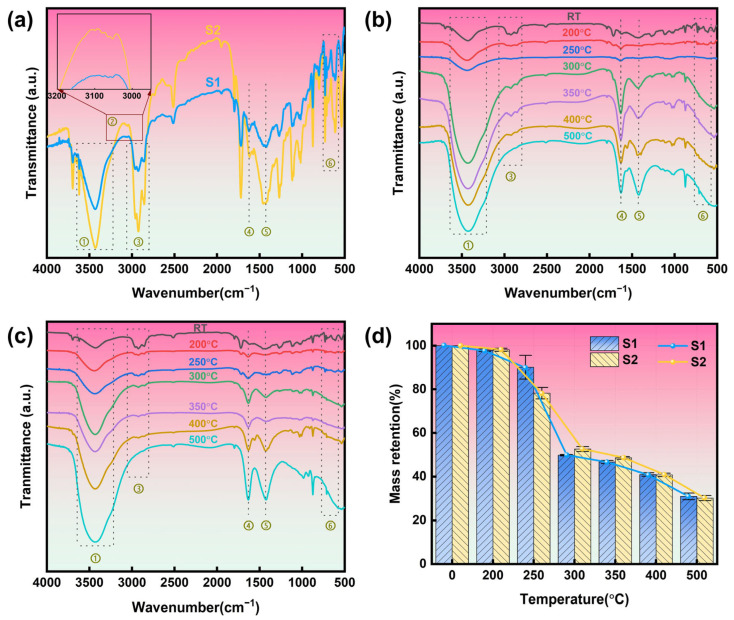
The FTIR spectra of cable sheath under different conditions: (**a**) unaged and aged materials at room temperature, (**b**) S1 in the muffle furnace test, (**c**) S2 in the muffle furnace test, and (**d**) unaged and aged materials mass retention at different temperatures.

Figure 7a presents the FTIR spectra of S1 and S2 at ambient temperature, and the FTIR results for S1 and S2 following six temperature gradient heat treatments in a muffle furnace are depicted in Figure 7b,c. The functional groups associated with the principal characteristic peaks are enumerated in Table 4 [4,12,42,43].

As illustrated in Figure 7a, S1 exhibits a pronounced characteristic peak corresponding to the stretching vibration of the C-Cl bond (C-Cl, *ν*) within the range of 570–740 cm^−1^. In contrast, the absorption intensity of S2 in this region markedly increased. Furthermore, the methylene in-plane bending vibration peak (CH_2_, δ) at 1425 cm^−1^, the methylene symmetric stretching vibration peak (CH_2_, νs) at 2856 cm^−1^, and the methylene antisymmetric stretching vibration peak (CH_2_, νas) at 2925 cm^−1^ for S2 all demonstrate a trend of heightened peak intensity and reduced peak width. This observation suggests that, following aging, the -CH_2_- structure of the PVC main chain retains a significant degree of integrity, which is potentially attributable to the formation of a cross-linked network that effectively mitigates the degradation of the main chain [20]. Notably, the characteristic peak of the conjugated double bond (C=C, ν) for S2 is observed at 1620 cm^−1^ [44]. Concurrently, the absorption peak of S2 in the range of 3040–3110 cm^−1^ is intensified, suggesting that aging has facilitated the emergence of unsaturated olefins (C=C-H), thereby further implying that de-chlorination reactions have transpired during the aging process. It is concluded that during the thermal aging stage, the primary changes in the cable structure are associated with the more reactive C-Cl bonds within the polymer molecule [44].

Analysis of Figure 7b,c reveals that the intensity of the hydroxyl stretching vibration peak (O-H, ν) for S1 and S2, observed within the range of 3220–3640 cm^−1^, exhibits a continuous increase with rising temperature. Concurrently, the absorption intensity in the spectral region of 2800–3060 cm^−1^ diminishes progressively, ultimately vanishing as the temperature escalates. This phenomenon is intricately linked to the advancement of the de-chlorination reaction. The simultaneous enhancement of the peak intensity associated with conjugated double bonds at 1620 cm^−1^ suggests that elevated temperatures facilitate the reaction in PVC, leading to the gradual degradation of the main chain -CH_2_- structure and the formation of additional C=C bonds.

The results of the muffle furnace test (Figure 7d) indicate that S2 undergoes decomposition at a faster rate than S1 within the temperature range of RT to 250 °C. Between 250 °C and 300 °C, both cable sheaths experience a significant mass loss, with S1 exhibiting a more rapid decomposition rate. Notably, a considerable amount of irritating gas (i.e., HCl) is released during this stage. In the temperature range of 300 °C to 500 °C, the release of the gas diminishes, and the pyrolysis rate of S2 slightly surpasses that of S1.

In summary, the analysis indicates that the de-chlorination reaction occurs during the thermal aging process. Furthermore, the increase in temperature accelerates the reaction, leading to the progressive disintegration of the main chain -CH_2_- structure and the formation of C=C bonds. The muffle furnace test results further demonstrate that S2 exhibits a slower pyrolysis rate at lower temperatures (below 250 °C), while its pyrolysis rate increases at higher temperatures (300–500 °C).

## 4. Conclusions

In this study, the effects of thermal aging on the surface morphology, pyrolysis, and combustion behavior of 110 kV flame-retardant cables were comprehensively evaluated using various methods, including SEM, TGA, LOI, UL-94, open flame, cone calorimeter tests. The results of SEM and CA tests showed that the roughness of the thermally aged cable sheath increased significantly, and protrusions, holes, and cracks appear. LOI, UL-94, and open flame tests indicated that the fire retardancy of the aged cables decreased, resulting in a higher fire hazard. The results of the cone calorimeter test indicated that the thermal aging treatment extended the ignition time of the cable sheath and reduced the HRR, THR, and FIGRA of the cable sheath. This suggests that after undergoing thermal aging, the cable sheath presents a lower fire hazard during forced combustion. Additionally, the TGA and muffle furnace tests revealed that the thermal aging treatment decreased the initial degradation temperature of the cable sheath. However, a greater yield of residual char was produced at elevated temperatures (700 °C), which can be attributed to the thermal oxidative decomposition of the aged cable sheath. This process resulted in the formation of a cross-linked structure, thereby enhancing thermal stability at high temperatures. In summary, this study examined the pyrolysis and fire retardancy of cable sheaths under both open and forced fire conditions. It analyzed the underlying causes, clarified the aging mechanisms, and provided valuable insights into cable fire safety. Additionally, it offered a reference for assessing fire risks associated with aging cables.

## Figures and Tables

**Table 1:** The results of the LOI and UL-94 tests (t_1_: the burning time of the sample after the first ignition; t_2_: the burning time of the sample after the second ignition).

Sample	t_1_ [s]	t_2_ [s]	LOI [%]	Dripping	Rating
S1	0	0	28.5	No	V-0
S2	3.5	0	28.3	No	V-0

**Table 2:** Cone calorimeter characteristic parameters of cable sheaths before and after thermal aging.

Sample	TTI [s]	pHRR1 [kW/m^2^]	pHRR2 [kW/m^2^]	THR [MJ/m^2^]	PSPR1 [m^2^/s]	PSPR2 [m^2^/s]	TSP [m^2^]	FIGRA [kW/(m^2^ s)]	MARHE [kW/m^2^]
S1	6	162.1	100.5	163.6	0.12	0.05	76.7	1.33	0.11
S2	8	133.7	122.6	123.7	0.08	0.05	41.7	0.92	0.10

**Table 3:** Pyrolysis characteristic parameters of unaged and aged cable sheaths.

Sample	T_5%_ [°C]	T_peak1_ [°C]	DTG_peak1_ [%min^−1^]	T_peak2_ [°C]	DTG_peak2_ [%min^−1^]	Char Residue [%]
S1	277.5	332.5	8.87	479.6	2.4	21.0
S2	271.5	329.2	7.86	481.8	2.1	23.9

**Table 4:** Functional groups of FTIR spectra of cable sheaths.

Number	Wavenumber (cm^−1^)	Functional Group
①	3220–3640	O-H stretching vibration
②	3040–3110	C=C-H
③	2800–3060	CH_2_
④	1620	C=C
⑤	1425	CH_2_
⑥	570–740	C-Cl

## Data Availability

The original contributions presented in the study are included in the article, further inquiries can be directed to the corresponding authors.
